# Piston Wear Detection and Feature Selection Based on Vibration Signals Using the Improved Spare Support Vector Machine for Axial Piston Pumps

**DOI:** 10.3390/ma15238504

**Published:** 2022-11-29

**Authors:** Shiqi Xia, Yimin Xia, Jiawei Xiang

**Affiliations:** 1 State Key Laboratory of High Performance Complex Manufacturing, Central South University, Changsha 410017, China; 2 College of Mechanical and Electrical Engineering, Wenzhou University, Wenzhou 325000, China

**Keywords:** piston wear, fault detection, feature selection, vibration signal, spare support vector machine, axial piston pump

## Abstract

A piston wear fault is a major failure mode of axial piston pumps, which may decrease their volumetric efficiency and service life. Although fault detection based on machine learning theory can achieve high accuracy, the performance mainly depends on the detection model and feature selection. Feature selection in learning has recently emerged as a crucial issue. Therefore, piston wear detection and feature selection are essential and urgent. In this paper, we propose a vibration signal-based methodology using the improved spare support vector machine, which can integrate the feature selection into the piston wear detection learning process. Forty features are defined to capture the piston wear signature in the time domain, frequency domain, and time–frequency domain. The relevance and impact of sparsity in 40 features are illustrated through the single and multiple statistical feature analysis. Model performance is assessed and the sparse features are discovered. The maximum model testing and training accuracy are 97.50% and 96.60%, respectively. Spare features *s*_10_, *s*_12_, *E*_w_(8), *x*_7_, *E*_e_(5), and *E*_e_(4) are selected and validated. Results show that the proposed methodology is applicable for piston wear detection and feature selection, with high model accuracy and good feature sparsity.

## 1. Introduction

**Figure 1:**
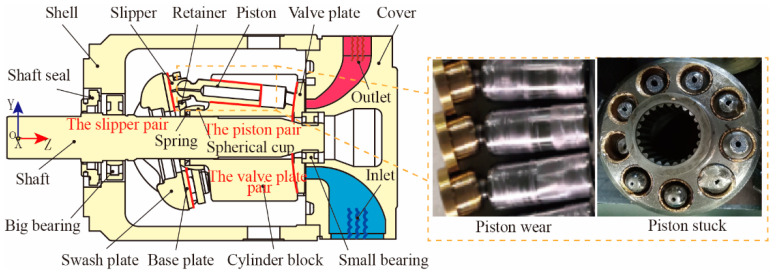
Typical structure and piston wear of axial piston pumps.

Hydraulic transmission systems have been widely utilized in the aviation, aerospace, and marine industries, with the advantages of high power density and output efficiency [1,2]. Axial piston pumps are the key power components in hydraulic systems, and they can convert the rotating mechanical power into fluid power [3,4,5]. Typical structures of an axial piston pump are shown in Figure 1. Pistons are uniformly distributed around the center of the cylinder block. The swash plate and retainer drive the pistons reciprocating along the cylinder block center. The oil suction and extrusion are accomplished through the reciprocating movements. There are three friction pairs acting as bearing and sealing functions (marked with red lines in Figure 1): the slipper pair, the piston pair, and the valve plate pair. The piston pair has poor bearing and sealing functions due to the huge lateral forces from the inclined swash plate [6,7,8]. The poor performance of the piston pair may lead the piston surface to easily wear and become stuck. The piston wear is caused by surface microprotrusion; different microstructures are proposed to improve the film characteristics of the piston pair [9]. Proper materials of friction matching can decrease the piston wear; 940 stainless steel, F102 engineering plastics, and Al_2_O_3_ ceramic were used as matching materials in a water hydraulic pump [10]. Ceramic and 30Cr2MoVA materials were applied to an ultra-high-pressure pump [11]. In addition, an anti-friction coating was added to high-lead bronze, and the mechanical properties and wear mechanism of the piston pair were evaluated. Uniform distributions of lead segregations can improve the piston wear properties [12]. Therefore, piston wear fault detection is of great significance for the system’s efficiency and lifetime. 

Numerous types of research have been carried out for the fault detection of an axial piston pump based on vibration signals. Methods based on cluster analysis [13], particle swarm optimization [14], artificial neural networks [15], hidden semi-Markov [16], and deep belief networks [17,18] are proposed, and features in the time domain (TD), frequency domain (FD), and time–frequency domains (TFD), including kurtosis, the root mean square, the energy ratio, the coefficients of the wavelet packet transform (WPT), and spectral entropy, are used as model indicators.

Although fault detection based on machine learning theory can achieve satisfactory accuracy, the performance is heavily dependent on model and feature selection [19]. Gaussian process regression (GPR), extreme gradient boosting, and support vector machine (SVM) are used for modeling the specific heat capacity in the application of solar energy [20]. Linear discriminant analysis, logistic regression, decision tree, random forest, Light-GBM, and multi-layer perceptron are utilized for the anomaly detection of the hydraulic system [21]. The GPR and support vector regression (SVR) are employed to predict the energy storage properties [22]. In addition, the convolutional neural network is proposed for the fault diagnosis of axial piston pumps [23]. Among the machine learning methods, SVM, with powerful generalization ability and high learning efficiency, has been widely used as a fault detection model for bearings [24,25,26], motors [27], gearboxes [28,29], and pumps [30]. Improved algorithms for SVM include the wavelet transform-based SVM [31], least squares-based SVM [32,33], hyper-sphere-structured SVM [34], proximal SVM [35], etc. In addition, feature selection in learning has recently emerged as a crucial issue [36,37]. Various forms of regularization are presented for feature selection during the machine learning process. The *ℓ*_2_ regularization is not enforced, and the results of the feature selection are poor. Numerous types of research based on the *ℓ*_1_ form, *ℓ*_1_/*ℓ*_2_ form, and *ℓ*_1_ + *ℓ*_2_ form have emerged to induce sparsity for feature selection in learning [38]. The *ℓ*_1_ regularization is widely utilized to select features during the machine learning process.

The model and feature selection during the fault detection of axial piston pumps are mostly based on expert knowledge. However, the relevance and impact of sparsity in model features are unclear. A piston wear fault is a major failure mode of axial piston pumps, but recent studies are not specific to piston wear detection and feature selection. The classical SVM is unable to complete feature selection during the learning process. In the presented study, a methodology using the improved SPARE-SVM is proposed for piston wear detection and feature selection for the first time. This methodology has the ability to integrate feature selection during the machine learning process. A large set of features is employed in the TD, FD, and TFD. The relevance and impact of sparsity in these features are illustrated through single and multiple statistical feature analysis, model performance evaluation, and feature selection analysis.

## 2. Materials and Methods

### 2.1. Experimental Setup and Procedure

**Figure 2:**
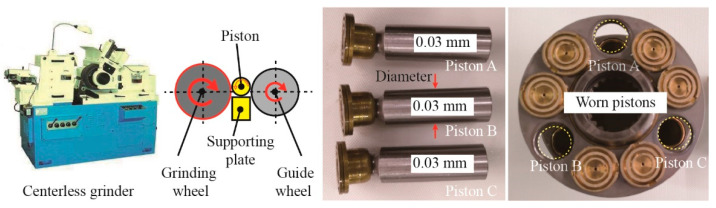
Process of the piston wear fault.

Testing pumps are axial piston pumps with swash-plate-type variable control. Detailed parameters are shown in Table 1. The testing pump has nine pistons. The materials of the piston pair are 38CrMoAl and CuPb15Sn5. The piston diameter is 17 mm. Large fitting clearance of the piston pair will increase the leakage, while small fitting clearance may lead to the piston becoming stuck. The normal fitting clearance between the piston and the cylinder for the testing pump is approximately 0.03 mm. A piston wear fault leads to the fitting clearance exceeding the level of tolerance. Abnormal vibrations will occur simultaneously. In this paper, additional clearance is processed through the centerless grinder to simulate the piston wear fault. As shown in Figure 2, the diameter of the three marked pistons (A, B, and C) is reduced by 0.03 mm.

**Figure 3:**
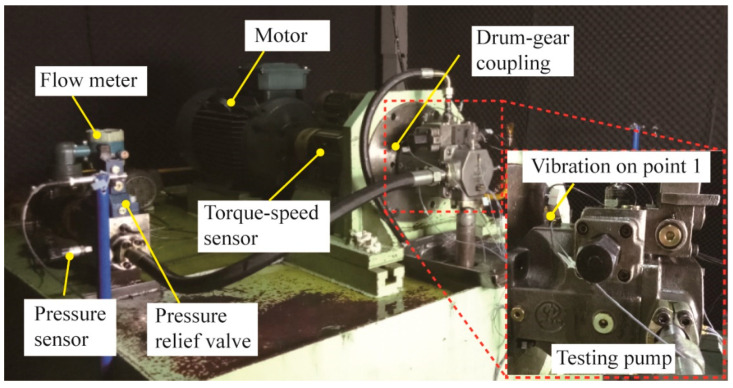
Layout of the test rig.

The testing rigs for the testing pump are shown in Figure 3. The testing pump is driven by an inverter-fed motor through the drum–gear coupling. A torque–speed transducer is used to monitor the torque and rotating speed, which is located between the testing pump and motor. The frequency converter can regulate the motor rotating speed. The discharge pressure is regulated by a pressure relief valve, and its maximum pressure is 35 MPa. The discharge pressure is measured by a pressure transducer and a pressure gauge. The pump output flow is measured by a flow transducer. The vibration signal is used for the piston wear fault detection and feature selection. The vibration acceleration on point 1 is collected by a piezoelectric accelerometer (Type LAN-XI 3050, Brüel & Kjær, Virum, Denmark) and data acquisition module (Type 4507-B-001, Brüel & Kjær, Virum, Denmark). The sampling frequency of vibration signals is 48,000 Hz. A high-pass filter is utilized to denoise raw vibration signals and the cut-off frequency is 7 Hz.

**Figure 4:**
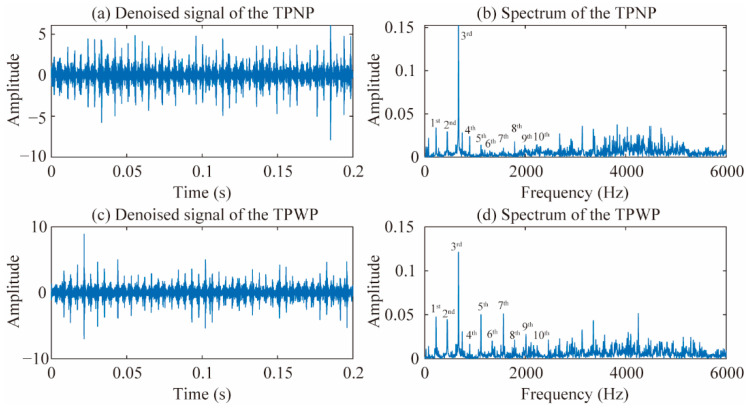
Denoised signals and the FT spectra. (**a**) Denoised signal of the TPNP; (**b**) spectrum of the TPNP; (**c**) denoised signal of the TPWP; (**d**) spectrum of the TPWP.

A testing pump with normal pistons (TPNP) and a testing pump with worn pistons (TPWP) are tested in the test rig, respectively. Vibration signals are compared in the TD, FD, and TFD. The denoised signals of the vibration acceleration on point 1 for the TPNP and TPWP are shown in Figure 4a,c, respectively. There are 9600 points (0.2 s) of the waveform in the TD. It is difficult to distinguish differences in the TD waveforms in Figure 4a,c. The signal’s inherent nature in the FD can be disclosed by Fourier transform (FT) analysis. The FT can reflect the frequency components and distribution of the vibration signal in the FD. There will be some abnormal frequencies in the signal spectra when a mechanical fault occurs. Spectra in the FD of the TPNP and TPWP are shown in Figure 4b,d, respectively. The frequency ranges from 0 Hz to 6000 Hz. Some differences in the spectrum distributions are presented. The amplitude of different harmonics is variable. 

**Figure 5:**
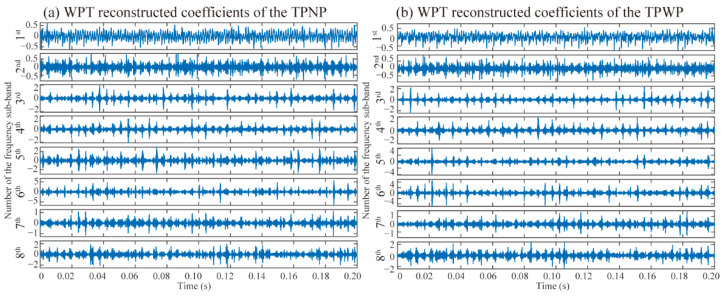
WPT-reconstructed coefficients. (**a**) TPNP; (**b**) TPWP.

Signal comparison in the TD and FD is based on stationary signals. The frequency spectrum obtained from the FT may fluctuate with time, because the mechanical fault signal is not stationary when a fault occurs. Signal processing methods in the TFD have been proposed to analyze the non-stationary signals. These methods include the short-time Fourier transform, Wigner–Ville distribution, wavelet transform (WT), WPT, empirical mode decomposition (EMD), and ensemble empirical mode decomposition (EEMD), etc. The WT or WPT decomposes a signal into an approximated component and a detailed component. Moreover, the WPT has high-frequency resolution power in both the low and high frequency range, because this transform can choose the matched frequency band adaptively. A signal can be divided into a series of IMFs and a residue by the EMD or EEMD algorithm. Furthermore, the EEMD algorithm is able to overcome the mode mixing issue by adding white noise with a finite amplitude and obtain better performance. Therefore, apart from signal comparisons in the TD and FD, TFD analyses based on the WPT and EEMD algorithms are conducted on the denoised vibration signals. The wavelet is sym4 and the decomposition level is 3 for the WPT algorithm. The WPT-reconstructed coefficients of the TPNP and TPWP are shown in Figure 5. The maximum amplitude of the fifth frequency sub-band of the TPNP is approximately 2, while the maximum amplitude of the fifth frequency sub-band of the TPWP is approximately 4. The maximum amplitude of the sixth frequency sub-band of the TPNP is approximately 5, while the maximum amplitude of the sixth frequency sub-band of the TPWP is approximately 4. In addition, there are also some differences in the waveforms of the WPT-reconstructed coefficients.

**Figure 6:**
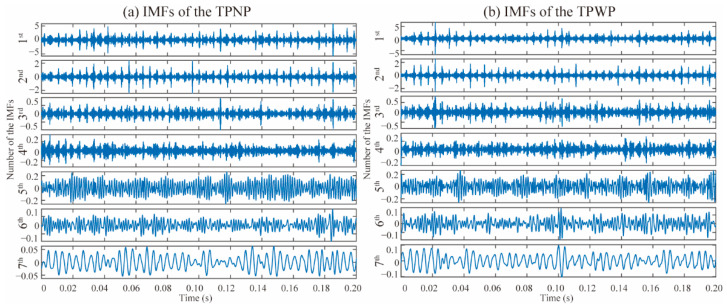
EEMD IMFs. (**a**) TPNP; (**b**) TPWP.

For the EEMD algorithm, the ratio of the standard deviation for the added sample noise is 0.2. The ensemble number is 50 and the IMF number is 7. The EEMD IMFs of the TPNP and TPWP are shown in Figure 6. The maximum amplitude of the seventh IMF of the TPNP is approximately 0.05, while the maximum amplitude of the seventh IMF of the TPWP is approximately 0.1. In addition, there are also some differences in the waveforms of the IMFs.

Results show that some differences in the TD, FD, and TFD can be found when the pump has a piston wear fault. These differences are caused by the waveform distortions and spectrum energy relocation. Therefore, it is feasible to detect the piston wear fault through the vibration signal. This paper proposes an improved SPARE-SVM-based methodology for the piston wear detection and feature selection of axial piston pumps. The training dataset contains 220 samples of the TPNP and 45 samples of the TPWP, and the testing dataset includes 30 samples of the TPNP and 10 samples of the TPWP. The length of a single sample is 512 data points. The training and testing samples are independent of each other.

### 2.2. Improved SPARE-SVM Based Methodology

The SVM algorithm has been widely used in various applications, with the advantages of uncomplicated principles, easy calculation, and high classification accuracy. This algorithm aims to construct a separating hyper-plane. The hyper-plane is able to divide the dataset (*x_i_*, *i =* 1, 2, ∙∙∙, *N*) into two classes (*y_i_* = ±1). The maximum distance between the upper and lower boundary of the hyper-plane is called the maximized margin. The support vectors are data points located on the hyper-plane. The separating hyper-plane of the SVM algorithm is constructed by structural risk minimization [25].

(1) $$f(x)=\omega^{T}x+b.$$

The optimal hyper-plane is built from parameters (*ω*, *b*), and estimations of the parameters are calculated as

(2) $$\left(\hat{\omega },\hat{b}\right)\in \arg\min\frac{1}{2}{\left\|\omega \right\|}_{2}^{2}+C\sum_{n=1}^{N}H_{\omega ,b}\left(x_{i},y_{i}\right),$$

where parameter *ω* is an *N*-dimensional vector and parameter *b* is a scalar. ‖*ω*‖_2_ is the *ℓ*_2_ form of the parameter *ω*. *H*(~) represents the hinge loss function of the *i*^th^ data (*x_i_*, *y_i_*). The weight of *H*(~) is adjusted by parameter *C*.

(3) $$H_{\omega ,b}\left(x_{i},y_{i}\right)=\max\{0,1-y_{i}\left(\omega^{T}x_{i}+b\right)\},$$

The classical SVM algorithm is unable to complete the feature selection during the machine learning process, due to the fact that the *ℓ*_2_ form of the parameter *ω* is used. In addition, imbalanced datasets of two classes (*y_i_* = ±1) may also decrease the parameter sparsity and model accuracy. Therefore, the improved SPARE-SVM algorithm is put forward, using the *ℓ*_1_ regularization form, the square hinge loss function *H*^2^(~), and the weights of the imbalanced datasets [39]. This algorithm can integrate the feature selection into the fault detection learning process.

The *ℓ*_1_ norm can impose the sparsity of parameter *ω*. Meanwhile, it renders the solution of the optimal hyper-plane difficult as the *ℓ*_1_ norm is not differentiable. The square hinge loss function *H*^2^(~) is able to deal with the non-differentiable problem of the *ℓ*_1_ norm and solve the optimal hyper-plane. The weights of the imbalanced datasets are imposed to increase the robustness and precision of the models. Parameter estimations of the improved SPARE-SVM algorithm are calculated as follows:

(4) $$\left(\hat{\omega },\hat{b}\right)\in \arg\min{\left\|\omega \right\|}_{1}+C\sum_{n=1}^{N_{-1}}H_{\omega ,b}^{2}\left(x_{i},y_{i}\right)+C\frac{N_{-1}}{N_{+1}}\sum_{n=1}^{N_{+1}}H_{\omega ,b}^{2}\left(x_{i},y_{i}\right),$$

where *N*_−1_ is the number of the class (*yi* = −1) and *N*_+1_ refers to the number of the other class (*yi* = +1).

The *ℓ*_1_ regularization norm of parameter *ω* is non-differentiable. The forward–backward splitting algorithm can solve this optimization problem using the gradient descent step of the square hinge loss function *H*^2^(~). The proximity operator of parameter *ω* is defined as

(5) $$\mathrm{Prox}\left\{\ell_{1}\mathrm{norm}\left[\omega \left(\ell_{1}\right)\right]\right\}=\mathrm{argmin}\frac{1}{2}{\left\|\omega \left(\ell_{1}\right)-\omega \right\|}_{2}^{2}+\ell_{1}\mathrm{norm}\left[\omega \left(\ell_{1}\right)\right],$$

where *ω*(*ℓ*_1_) is the parameter estimation of the *ℓ*_1_ regularization norm. The parameter *ω* is obtained by the iterative calculation of the forward-backward splitting algorithm.

(6) $$\omega \left(z\right)=\mathrm{Prox}\left\{\omega \left(z-1\right)-\frac{1}{D}\nabla H_{\omega \left(z-1\right),b}^{2}\left(x_{i},y_{i}\right)\right\},$$

where *ω*(*z*) and *ω*(*z* − 1) are the parameter estimations of the *z*th and *z* − 1th iterative. The parameter *D* is a constant of gradient descent order, and ∇*H*^2^(~) refers to the Lipschitz-continuous gradient of the square hinge loss function.

**Figure 7:**
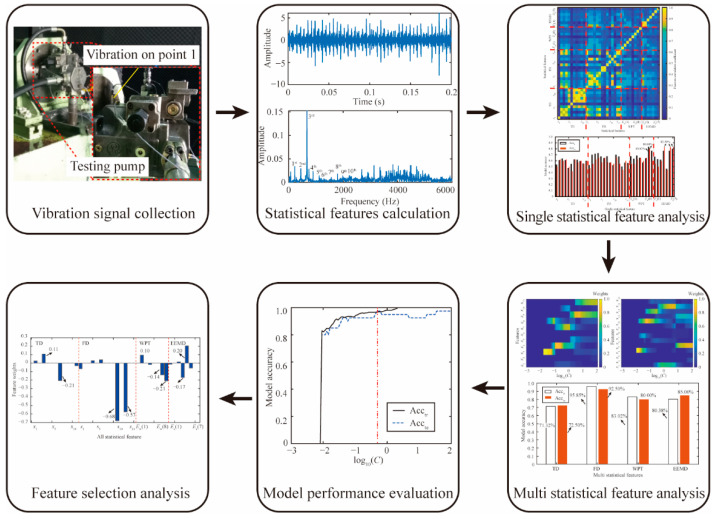
Flowchart of the improved SPARE-SVM-based methodology.

The improved SPARE-SVM-based methodology is proposed for the piston wear detection and feature selection of axial piston pumps. The flowchart of this methodology is shown in Figure 7. First, the vibration signals of the axial piston pump are collected by the data acquisition module. Second, a comprehensive set of features is defined in the TD, FD, and TFD. Third, single statistical feature analysis is conducted to investigate the feature correlations and model performance obtained from a single feature. Fourth, multiple statistical feature analysis is used to illustrate the weight variations and model performance of multiple features in the TD, FD, and TFD. Fifth, the weight variations and model accuracy of the large set of features are analyzed through the model performance evaluation. Lastly, feature selection analysis is utilized to validate the feature sparsity.

Differences in the waveforms in the TD, FD, and TFD are shown through the signal comparison in Section 2.1. Statistical feature calculation in the TD [40,41], FD [42], and TFD [43,44] is conducted, and 40 features are constructed to capture the fault characteristics from different aspects. 

Statistical features used in the TD are listed in Table 2, where *x_i_* (*i* = 1, 2, …, 11) denotes the *i*^th^ feature, *x*(*n*) (*n* = 1, 2, …, *N*) refers to the TD signal, and *N* is the data length of the vibration signal. The mean value and standard deviation (*x*_1_ and *x*_2_) reflect the average and deviation of the TD signal. The root amplitude, root mean square, and peak value (*x*_3_, *x*_4_, and *x*_5_) refer to the signal amplitude and energy. The skewness (*x*_6_) measures the data asymmetry around the mean value. The kurtosis (*x*_7_) shows the outlier proneness of a distribution. The crest factor (*x*_8_) and clearance factor (*x*_9_) indicate peak severity. The shape factor (*x*_10_) reveals the shape of the distribution. The impulse factor (*x*_11_) detects the impulse components of the distribution.

Statistical features in the FD are shown in Table 3, where *s_i_* (*i* = 1, 2, …, 14) denotes the *i*^th^ feature, *s*(*k*) (*k* = 1, 2, …, *K*) is the amplitude and *f*(*k*) (*k* = 1, 2, …, *K*) represents the corresponding frequency for the *k*th FT spectrum line, and *K* refers to the FT spectrum line number. The mean frequency (*s*_1_) reflects the signal energy in the FD. The frequency center, root 2 order weighting, root 4-2 order weighting, 2-4-order weighting, 2 order center moment, 3 order center moment, 4 order center moment, and root 2 order center moment (*s*_2_, *s*_3_, *s*_4_, *s*_5_, *s*_6_, *s*_7_, *s*_8_, and *s*_9_) refer to the main frequency position changes in the frequency spectrum. The root 2 order convergence index, 3 order convergence index, 4 order convergence index, 1/2 order convergence index, and root 2 order convergence index (*s*_10_, *s*_11_, *s*_12_, *s*_13_, and *s*_14_) reveal the convergence characteristics of the frequency spectrum power. 

Signal features obtained from the TFD are effective compensations for the statistical features in the TD and FD. The energy ratio of the frequency sub-bands decomposed by the WPT and the energy ratio of the IMFs decomposed by the EEMD are used as statistical features in the TFD. As shown in Table 4, *E*_w_(*i*) (*i* = 1, 2, …, 2*^m^*) denotes the *i*^th^ frequency sub-band feature of the WPT, *x_i_*(*j*) refers to the *i*^th^ frequency sub-band, and *m* represents the decomposition level (*m* = 3). For the EEMD algorithm, *E*_e_(*i*) (*i* = 1, 2, …, *L*) denotes the *i*^th^ IMF feature, *c_i_*(*j*) refers to the *i*^th^ IMF, and *L* represents the number of IMFs (*L* = 7). 

## 3. Results and Discussion

### 3.1. Single Statistical Feature Analysis

**Figure 8:**
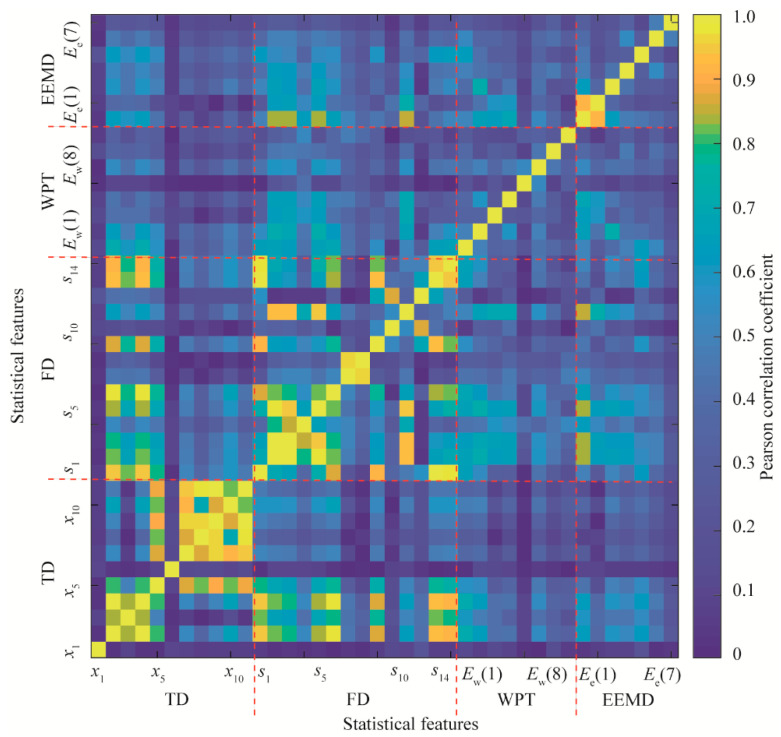
Correlations between statistical features.

Statistical feature correlations and model performance obtained from the single feature were investigated via single statistical feature analysis. The correlation analysis of the features of all training samples was carried out. The absolute values of the Pearson correlation coefficients (PCCs) are shown in Figure 8. The diagonal entries are only 1 due to the fact that all statistical features are always directly correlated to themselves. Statistical features in the TD or FD have a strong correlation. For example, the root mean square (*x*_4_) is directly correlated to the standard deviation (*x*_2_) as the PCC is 1. The PCCs between *x*_9_ and *x*_8_, *x*_7_ are 0.954 and 0.959, respectively. The PCCs between *x*_11_ and *x*_9_, *x*_8_, *x*_7_ are 0.996, 0.977, and 0.946, respectively. 

In terms of the features in the FD, the PCC between *s*_3_ and *s*_2_ is 0.993. The PCCs between *s*_5_ and *s*_3_, *s*_2_ equal 0.952 and 0.977, respectively. The PCC between *s*_8_ and *s*_7_ is 0.954. The PCC between *s*_9_ and *s*_1_ equals 0.920. The PCCs between *s*_11_ and *s*_5_, *s*_3_, *s*_2_ are 0.940, 0.926, and 0.924, respectively. The PCCs between *s*_13_ and *s*_9_, *s*_1_ equal 0.933 and 0.986, respectively. The PCCs between *s*_14_ and *s*_13_, *s*_1_ are 0.951 and 0.970, respectively. 

Moreover, some statistical features in the FD are highly correlated to those in the TD. For instance, the PCCs between *s*_1_ and *x*_4_, *x*_2_ equal 0.943. The PCCs between *s*_6_ and *x*_4_, *x*_2_ equal 0.976. The PCCs between *s*_13_ and *x*_4_, *x*_2_ equal 0.930. The PCCs between *s*_14_ and *x*_4_, *x*_2_ equal 0.915. Strong correlations are due to the fact that the calculation processes of some statistical features in the TD and FD are related to each other. 

In terms of statistical features in the TFD, most features calculated by the WPT and EEMD have a lower correlation with each other due to frequency decompositions of the vibration signals. Therefore, statistical features with lower correlations in the TFD have better independence than features in the TD or FD.

**Figure 9:**
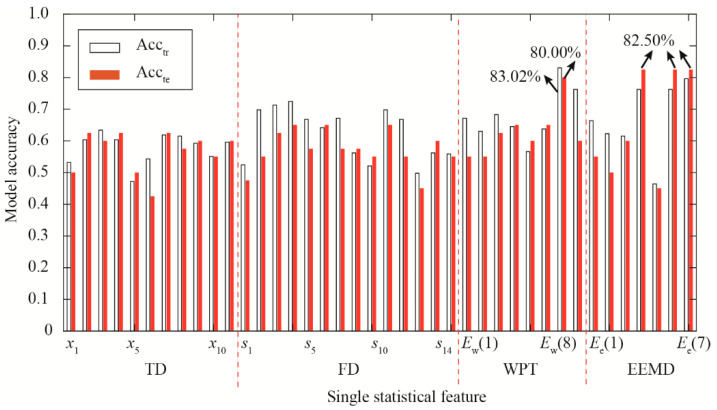
Model performance obtained from single statistical feature.

A single statistical feature is utilized as a model indicator for piston wear detection. The model accuracy of the training dataset (Acc_tr_) and the testing dataset (Acc_te_) is shown in Figure 9. Features *E*_e_(4), *E*_e_(6), and *E*_e_(7) in the TFD yield better testing performance, with Acc_te_ of 82.50%. Feature *E*_w_(7) has the best training performance, with the maximum Acc_tr_ of 83.02%. Moreover, the Acc_te_ of this single feature equals 80.00%. In addition, most features in the FD have better model accuracy than features in the TD. Features, *s*_2_, *s*_3_, *s*_4_, *s*_5_, *s*_6_, *s*_7_, *s*_10_, and *s*_11_ in the FD have larger Acc_tr_ values than features in the TD. A single feature of the energy ratio is able to capture the piston wear information. Results of the single statistical feature analysis show that a single feature in the TFD has better independence and model accuracy. 

### 3.2. Multiple Statistical Feature Analysis

**Figure 10:**
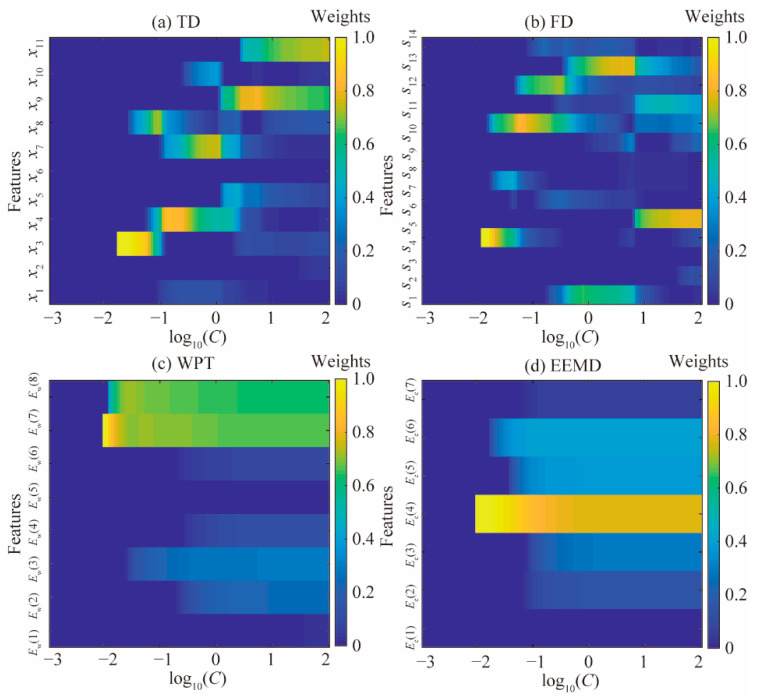
Weight variations of multiple features. (**a**) TD; (**b**) FD; (**c**) WPT; (**d**) EEMD.

Weight variations of multiple features in the TD, FD, and TFD are shown in Figure 10. Abscissas are the logarithmic coordinates of parameter *C*. The parameter *C* ranges from 1.00 × 10^−3^ to 1.00 × 10^2^, and it can adjust the weights of the square hinge loss function *H*^2^(~). Vertical coordinates refer to feature weights varying with parameter *C* during the piston wear detection process. Figure 10a shows that features *x*_3_, *x*_4_, *x*_7_, and *x*_8_ are the four first features selected by the multiple statistical feature analysis using the improved SPARE-SVM algorithm. These features are excluded as *C* increases. For features (*x*_7_, *x*_8_, *x*_9_, and *x*_11_), with strong correlations in the TD, only features *x*_9_ and *x*_11_ remain included when increasing parameter *C*. As shown in Figure 10b, features *s*_5_, *s*_11_, and *s*_13_ are selected among the multiple correlated features in the FD. Weight variations of multiple features calculated by the WPT are shown in Figure 10c. *E*_w_(7) and *E*_w_(8) are selected as the two first features and they remain included during the piston wear detection process. The three first selected features from the EEMD are *E*_e_(4), *E*_e_(5), and *E*_e_(6), as shown in Figure 10d, and they continue to be selected as parameter *C* increases.

**Figure 11:**
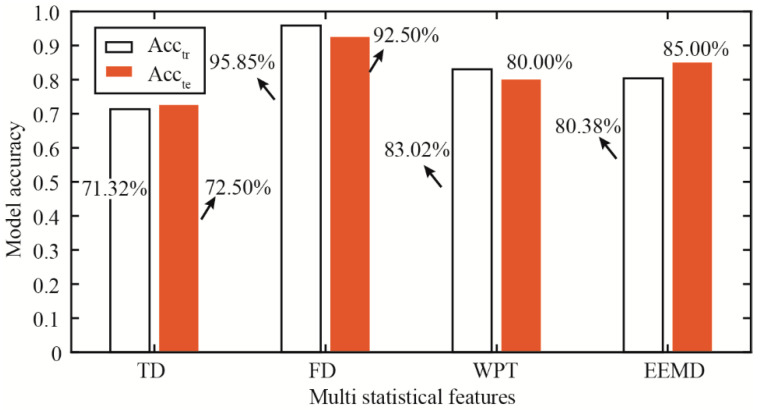
Model performance obtained from multiple statistical features.

Multiple statistical features calculated from the TD, FD, WPT, and EEMD are employed as model indicators for piston wear detection, respectively. Moreover, the model accuracy Acc_tr_ and Acc_te_ is shown in Figure 11. Multiple features in the FD have the maximum model accuracy, where Acc_tr_ and Acc_te_ are 95.85% and 92.50%, respectively. Features in the TD have the minimum model accuracy, where Acc_tr_ and Acc_te_ are 71.32% and 72.50%, respectively. The Acc_tr_ and Acc_te_ for the WPT are 83.02% and 80.00%, respectively. Moreover, the Acc_tr_ and Acc_te_ for the EEMD are 80.38% and 85.00%, respectively. The model accuracy of multiple statistical features in the TFD is larger than that of features in the TD. Results show that piston wear detection based on multiple statistical features in the TD is not applicable. Features in the FD and TFD are effective compensations for the detection model. The model accuracy of features in the FD and TFD is higher and satisfactory. 

### 3.3. Model Performance Evaluation

**Figure 12:**
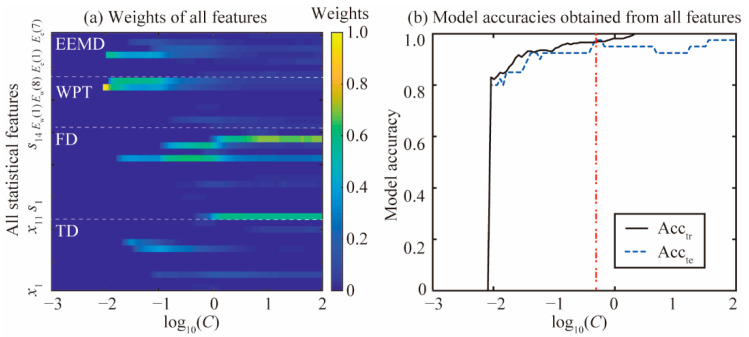
Weight variations and model accuracy for all features. (**a**) Weight variations of all features; (**b**) model accuracy obtained from all features.

The large set of features in the TD, FD, and TFD is used as the input of the improved SPARE-SVM model for piston wear detection simultaneously. All feature weights varying with parameter *C* during the model learning process are shown in Figure 12a. All statistical features are excluded when parameter *C* is smaller than 9.11 × 10^−3^. The energy ratio features *E*_w_(7), *E*_w_(8), and *E*_e_(4) are the three first features selected due to the high independence and model accuracy, and they are shown in Figure 8 and Figure 9, respectively. These variations are verified with the results of the multiple statistical feature analysis shown in Figure 10c,d. Most statistical features calculated from the TD, WPT, and EEMD have decreasing weights when parameter *C* continues to increase. The weights of some statistical features (*s*_1_, *s*_10_, *s*_12_, and *s*_13_) in the FD increase as parameter *C* increases. They agree with the results shown in Figure 10b.

The model accuracy Acc_tr_ and Acc_te_ varying with parameter *C* during the model learning process is shown in Figure 12b. There is a sudden change in the model accuracy when *C* is around 9.11 × 10^−3^. The change is due to the fact that a large set of features in the TD, FD, and TFD is not selected during the model learning process when *C* is less than 9.11 × 10^−3^. The Acc_te_ increases as parameter *C* increases. The maximum value of the Acc_te_ of 97.50% occurs when *C* is around 4.75 × 10^−1^. Moreover, the Acc_tr_ is 96.60% at this time. The Acc_te_ is no longer improved as parameter *C* increases. In addition, when *C* is larger than 6.00 × 10^−1^, the Acc_te_ begins to decline. The model accuracy Acc_tr_ gradually increases as parameter *C* becomes larger, which means that over-fitting occurs in the model learning process. It is found that the parameter *C* is able to regulate the trade-off between the feature sparsity and model accuracy. Therefore, the optimal parameter *C* is chosen as 4.75 × 10^−1^ through the model performance evaluation. It is found that the model accuracy for all features is higher than that obtained from the single and multiple statistical feature analysis. This means that the model accuracy for all features is more satisfactory. 

### 3.4. Feature Selection Analysis

**Figure 13:**
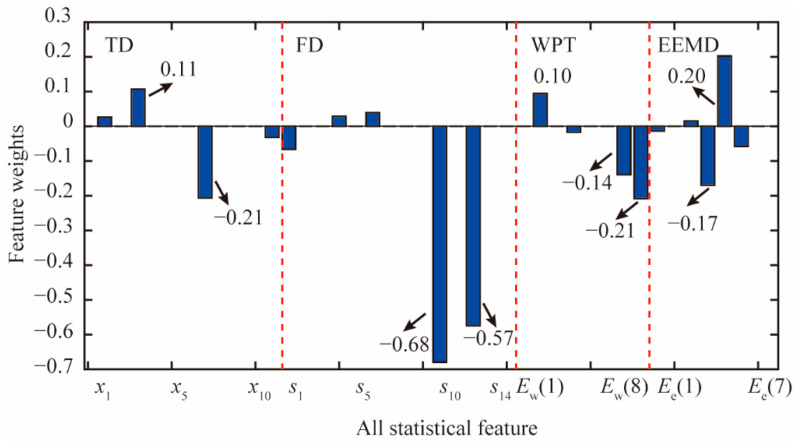
Weight variations of all features with *C* = 4.75 × 10^−1^.

Considering that the classical SVM algorithm cannot complete the feature selection during the machine learning process, the feature selection analysis is only conducted based on the improved SPARE SVM algorithm. The weights of the large set of features in the TD, FD, and TFD with *C* = 4.75 × 10^−1^ are shown in Figure 13. Statistical features *x*_1_, *x*_3_, *x*_7_, *x*_11_, *s*_1_, *s*_4_, *s*_6_, *s*_10_, *s*_12_, *E*_w_(2), *E*_w_(4), *E*_w_(7), *E*_w_(8), *E*_e_(1), *E*_e_(3), *E*_e_(4), *E*_e_(5), and *E*_e_(6) are selected by the improved SPARE-SVM algorithm. Meanwhile, features *x*_2_, *x*_4_, *x*_5_, *x*_6_, *x*_8_, *x*_9_, *x*_10_, *s*_2_, *s*_3_, *s*_5_, *s*_7_, *s*_8_, *s*_9_, *s*_11_, *s*_13_, *s*_14_, *E*_w_(1), *E*_w_(3), *E*_w_(5), *E*_w_(6), *E*_e_(2), and *E*_e_(7) are excluded with no weights. Features *s*_10_ and *s*_12_ in the FD have larger weights than other features. The weights of *s*_10_ and *s*_12_ are −0.68 and −0.57, respectively. The weights of the features *x*_3_ and *x*_7_ in the TD are 0.11 and −0.21, respectively. The weights of the features *E*_w_(2), *E*_w_(7), and *E*_w_(8) calculated by the WPT are 0.10, −0.14, and −0.21, respectively. The weights of the features *E*_e_(4) and *E*_e_(5) calculated by the EEMD are −0.17 and 0.20, respectively. The order of the first six sparse features is *s*_10_, *s*_12_, *E*_w_(8), *x*_7_, *E*_e_(5), and *E*_e_(4).

**Figure 14:**
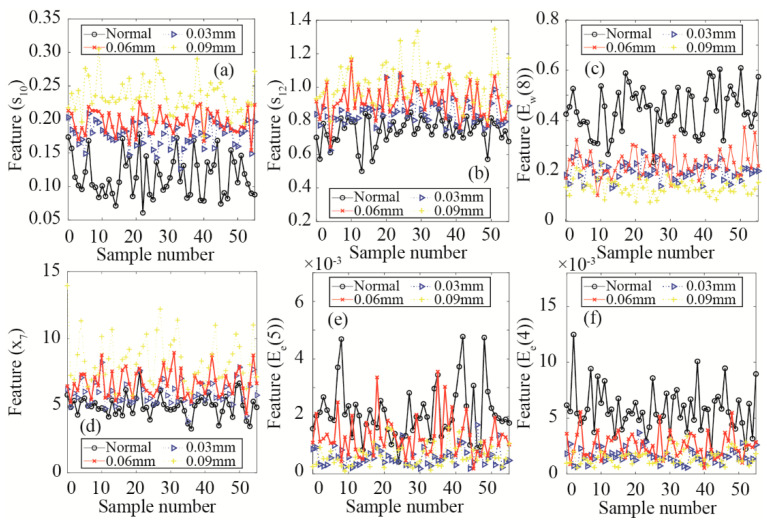
Six sparse feature comparisons under different cases of piston wear.

Different cases of additional clearance (0.03 mm, 0.06 mm, and 0.09 mm) are investigated to validate the six sparse features selected by the improved SPARE-SVM algorithm for piston wear detection. Feature comparisons under different cases of piston wear are shown in Figure 14. The abscissa is the sample number, and the sparse features of 55 samples are calculated. As shown in Figure 14a,b, different wear cases affect the convergence characteristics of the frequency spectrum power, especially for the root 2 order convergence index (*s*_10_) and 4 order convergence index (*s*_12_,). The indexes *s*_10_ and *s*_12_ become larger as the clearance increases. The case of the normal piston can be identified from the energy ratio features *E*_w_(8), *E*_e_(5), and *E*_e_(4), as shown in Figure 14c,e,f, respectively. The case of 0.09 mm is different from the other three cases in terms of kurtosis (*x*_7_). The six major sparse features of the piston wear fault are *s*_10_, *s*_12_, *E*_w_(8), *x*_7_, *E*_e_(5), and *E*_e_(4) due to the weight differences. Results show that the improved SPARE-SVM model is effective for piston wear fault detection and sparse feature selection.

## 4. Conclusions

This paper proposes a methodology using the improved SPARE-SVM for piston wear detection and feature selection for the first time. An experimental investigation was carried out, and the following conclusions were drawn.

1. The proposed methodology can integrate feature selection during the machine learning process of piston wear detection.
2. A large set of features is constructed in the TD, FD, and TFD. Single and multiple feature analysis are utilized to illustrate the relevance and impact of sparsity in the comprehensive set of features.
3. Feature effects on the model accuracy are analyzed. The maximum model testing and training accuracy values are 97.50% and 96.60%, respectively.
4. Spare features *s*_10_, *s*_12_, *E*_w_(8), *x*_7_, *E*_e_(5), and *E*_e_(4) are selected and validated.

Future work will focus on the improvement of the SPARE-SVM algorithm. Then, this improved algorithm will be used for other types of fault detection in axial piston pumps. 

## Figures and Tables

**Table 1:** Detailed descriptions of the testing pumps.

No.	Parameters	Values
1	Rated speed	1500 r/min
2	Maximum value of the displacement	40 cm^3^/r
3	Maximum value of the discharge pressure	35 MPa
4	Piston diameter	17 mm
5	Piston number	9

**Table 2:** Statistical features in the TD.

No.	Description	Expression	No.	Description	Expression
1	Mean value	$x_{1}=\frac{1}{N}\sum_{n=1}^{N}x\left(n\right)$	2	Standard deviation	$x_{2}=\sqrt{\frac{1}{N-1}\sum_{n=1}^{N}{\left[x\left(n\right)-x_{1}\right]}^{2}}$
3	Root amplitude	$x_{3}={\left[\frac{1}{N}\sum_{n=1}^{N}\sqrt{\left|x\left(n\right)\right|}\right]}^{2}$	4	Root mean square	$x_{4}=\sqrt{\frac{1}{N}\sum_{n=1}^{N}x{\left(n\right)}^{2}}$
5	Peak value	$x_{5}=\max\left|x\left(n\right)\right|$	6	Skewness value	$x_{6}=\frac{1}{\left(N-1\right)x_{2}^{3}}\sum_{n=1}^{N}{\left[x\left(n\right)-x_{1}\right]}^{3}$
7	Kurtosis value	$x_{7}=\frac{1}{\left(N-1\right)x_{2}^{4}}\sum_{n=1}^{N}{\left[x\left(n\right)-x_{1}\right]}^{4}$	8	Crest factor	$x_{8}=\frac{x_{5}}{x_{4}}$
9	Clearance factor	$x_{9}=\frac{x_{5}}{x_{3}}$	10	Shape factor	$x_{10}=Nx_{4}/\sum_{n=1}^{N}\left|x\left(n\right)\right|$
11	Impulse factor	$x_{11}=Nx_{5}/\sum_{n=1}^{N}\left|x\left(n\right)\right|$			

**Table 3:** Statistical features in the FD.

No.	Description	Expression	No.	Description	Expression
1	Mean frequency	$s_{1}=\frac{1}{K}\sum_{k=1}^{K}s\left(k\right)$	2	Frequency center	$s_{2}=\sum_{k=1}^{K}f\left(k\right)s\left(k\right)/\sum_{k=1}^{K}s\left(k\right)$
3	Root 2 order weighting	$s_{3}=\sqrt{\sum_{k=1}^{K}f^{2}\left(k\right)s\left(k\right)/\sum_{k=1}^{K}s\left(k\right)}$	4	Root 4-2 order weighting	$s_{4}=\sqrt{\sum_{k=1}^{K}f^{4}\left(k\right)s\left(k\right)/\sum_{k=1}^{K}f^{2}\left(k\right)s\left(k\right)}$
5	2-4 order weighting	$s_{5}=\sum_{k=1}^{K}f^{2}\left(k\right)s\left(k\right)/\sqrt{\sum_{k=1}^{K}f^{4}\left(k\right)s\left(k\right)\sum_{k=1}^{K}s\left(k\right)}$	6	2 order center moment	$s_{6}=\frac{1}{K-1}\sum_{k=1}^{K}{\left[s\left(k\right)-s_{1}\right]}^{2}$
7	3 order center moment	$s_{7}=\sum_{k=1}^{K}{\left[s\left(k\right)-s_{1}\right]}^{3}/K{\sqrt{s_{6}}}^{3}$	8	4 order center moment	$s_{8}=\sum_{k=1}^{K}{\left[s\left(k\right)-s_{1}\right]}^{4}/Ks_{6}^{2}$
9	Root 2 order center moment	$s_{9}=\sqrt{\frac{1}{K}\sum_{k=1}^{K}{\left[f\left(k\right)-s_{2}\right]}^{2}s\left(k\right)}$	10	Root 2 order center moment convergence index	$s_{10}=s_{9}/s_{2}$
11	3 order convergence index	$s_{11}=\frac{1}{Ks_{9}^{3}}\sum_{k=1}^{K}{\left[f\left(k\right)-s_{2}\right]}^{3}s\left(k\right)$	12	4 order convergence index	$s_{12}=\frac{1}{Ks_{9}^{4}}\sum_{k=1}^{K}{\left[f\left(k\right)-s_{2}\right]}^{4}s\left(k\right)$
13	1/2 order convergence index	$s_{13}=\frac{1}{K\sqrt{s_{9}}}\sum_{k=1}^{K}{\left|f\left(k\right)-s_{2}\right|}^{1/2}s\left(k\right)$	14	Root 2 order convergence index	$s_{14}=\sqrt{\sum_{k=1}^{K}{\left[f\left(k\right)-s_{2}\right]}^{2}s\left(k\right)/\sum_{k=1}^{K}s\left(k\right)}$

**Table 4:** Statistical features in the TFD.

No.	Description	Expression
1	Frequency band energy ratio	$E_{w}\left(i\right)=\sum_{j=1}^{2^{-m}N}{\left[x_{i}\left(j\right)\right]}^{2}/\sum_{i=1}^{2^{m}}\sum_{j=1}^{2^{-m}N}{\left[x_{i}\left(j\right)\right]}^{2},i=1,2,\ldots ,2^{m}$
2	IMF energy ratio	$E_{e}\left(i\right)=\sum_{j=1}^{N}{\left[c_{i}\left(j\right)\right]}^{2}/\sum_{i=1}^{L}\sum_{j=1}^{N}{\left[c_{i}\left(j\right)\right]}^{2},i=1,2,\ldots ,L$

## Data Availability

The data presented in this study are available on request from the corresponding author. The data are not publicly available due to an ongoing study.
